# Adaptations to In-Home Health Care Due to COVID-19: The VA’s Home-Based Primary Care Program

**DOI:** 10.1093/geroni/igab046.1798

**Published:** 2021-12-17

**Authors:** Tamar Wyte-Lake, Leah Haverhals, Chelsea Manheim, Nelly Solorzano, Suzanne Gillespie

**Affiliations:** 1 US Department of Veterans Affairs, U.S. Department of Veterans Affairs (VA), California, United States; 2 Denver Center of Innovation for Veteran Centered and Value Driven Care (COIN), Denver, Colorado, United States; 3 Finger Lakes VA Healthcare System, Canandaigua, New York, United States

## Abstract

The COVID-19 pandemic disrupted traditional Home Based Primary Care (HBPC) care processes, including changes to provision of face-to-face care in-home for older adults. Our study describes and explains care delivery changes Department of Veterans Affairs (VA) HBPC programs made in response to the pandemic. We fielded a national survey to all 140 VA HBPC programs, targeting interdisciplinary care teams and HBPC leadership. We structured survey questions using a mixed method approach with both closed and open-ended questions, applying a qualitative content analysis approach to open-ended responses complemented by analysis of descriptive quantitative data. Preliminary findings highlight the value and consideration of different telehealth modalities when caring for an older, homebound population, as well as creative adaptations HBPC teams made to deliver care during the pandemic. Implications include nascent development of decision-making paradigms beyond the pandemic particularly for appropriate use of telehealth modalities for older homebound adults.

